# HOME-BASED TRANSCRANIAL DIRECT CURRENT STIMULATION AND CLINICAL PAIN IN OLDER ADULTS: A RANDOMIZED CLINICAL STUDY

**DOI:** 10.1093/geroni/igac059.1394

**Published:** 2022-12-20

**Authors:** Hyochol Ahn, Geraldine Martorella, Kenneth Mathis, Hongyu Miao

**Affiliations:** Florida State University, Tallahassee, Florida, United States; Florida State University, Tallahassee, Florida, United States; University of Texas Health Science Center at Houston, Houston, Florida, United States; Florida State University, Tallahassee, Florida, United States

## Abstract

Knee osteoarthritis (OA) is one of the leading causes of pain in older adults. Previous studies indicated clinic-based transcranial direct current stimulation (tDCS) was effective to reduce pain in various populations, but no published studies have reported the efficacy of home-based self-administered tDCS in older adults with knee OA using randomized clinical study. Thus, the purpose of this study was to evaluate the efficacy and safety of tDCS on clinical pain intensity in adults with knee OA pain. One hundred twenty participants aged 50–85 years with knee OA pain were randomly assigned to receive fifteen daily sessions of 2 mA tDCS for 20 min (n = 60) or sham tDCS (n = 60) over 3 weeks with remote supervision via telehealth. Clinical pain intensity was measured by asking participants to rate their knee pain using a numeric rating scale from 0 (no pain) to 100 (worst pain imaginable). Participants (68% female) had a mean age of 66 years and the mean body mass index in the sample was 32.59 kg/m2. There have been no adverse events. Active tDCS significantly reduced pain intensity compared to sham tDCS after completion of the fifteen daily sessions (t = 6.57, df = 110, p < .001). We demonstrated that home-based self-administered tDCS was safe and reduced clinical pain intensity in older adults with knee OA, which can increase its accessibility. Future studies with multi-site randomized controlled trials with various populations are needed to validate our findings.

